# The Importance of Statistical Competencies for Medical Research Learners

**DOI:** 10.1080/10691898.2018.1484674

**Published:** 2018-08-21

**Authors:** Robert A. Oster, Felicity T. Enders

**Affiliations:** a Division of Preventive Medicine, Department of Medicine, University of Alabama at Birmingham, Birmingham, AL; b Division of Biomedical Statistics and Informatics, Department of Health Sciences Research, Mayo Clinic, Rochester, MN

**Keywords:** Assessment, Biostatistics, Research training, Statistical competency

## Abstract

It is very important for medical professionals and medical researchers to be literate in statistics. However, we have found that the degree of literacy that is required should not be identical for every statistical competency or even for every learner. We first begin by describing why the development, teaching, and assessment of statistical competencies for medical professionals and medical researchers are critical tasks. We next review our three substantial efforts at developing a comprehensive list of statistical competencies that can be used as a guide for what medical research learners should know about statistics, for curricular development, and for assessment of statistical education. We then summarize the origin and the inclusion of the statistical competency items. We follow this with a description of potential uses and applications of the statistical competencies to improve targeted learning for medical research learners. Finally, we discuss implications of the statistical competencies for undergraduate statistics education.

## 1. Introduction

In considering statistics education at the undergraduate and graduate level, it may be informative to consider how that education might be used by students who go on to become physicians or other health scientists. In this article, our goal is to inform the readers of this journal about recent developments in the field of statistics education for medical researchers and health scientists.

It is very important for medical researchers and health science learners to be literate in biostatistics, as biostatistics is frequently used to assist in the design of medical research studies and to summarize, analyze, and report on data obtained from these studies. In recent years, results obtained from statistical analyses have been reported in the medical literature on an increasing basis (Altman et al. 2002; Horton and Switzer 2005; Strasak et al. 2007), and so an increased knowledge of biostatistics may be needed in order to fully understand results that are being reported and translate those results to research or practice. Additional familiarity in biostatistics is also needed in order to understand when statistical methods have been incorrectly applied or when statistical results have been incorrectly reported; it has been noted that without the benefit of statistical expertise, statistical mistakes are common in manuscripts that have been submitted to or already published in peer-reviewed journals (Harris et al. 2009; Mazumdar et al. 2010; Fernandes-Taylor et al. 2011).

The development, teaching, and assessment of statistical competencies for medical research are essential tasks. Competency has been defined as the ability or skill to do something successfully or efficiently (Oxford Dictionary 2018). Statistical competencies are used to define statistical education in terms of the topics that should be taught and more generally for designing curricula for learners. Our previous and current work in this area is oriented toward the statistical education of medical research learners. In the remainder of this article, we will review our previous efforts, describe our current efforts, summarize the origin and the inclusion of statistical competency items, and discuss possible future directions toward the need for and development, assessment, implementation of statistical competencies for medical research learners, and implications of this work for undergraduate statistics education.

## 2. Need for A New Assessment Tool to Assess Statistical Competencies

A set of competencies was developed for Master’s degree-level training in clinical and translational science (CTS) by the Education Key Function Committee of the Clinical and Translational Science Award’s (CTSA) National Consortium (CTSA Education Core Competency Workgroup 2011). This set of competencies included ten statistical competencies in the core thematic area of statistical approaches. Separate from this effort, a core competency model for the Master of Public Health (PH) degree was developed (Calhoun et al. 2008). This model included ten statistical competencies in the biostatistics core domain.

Graduate-level statistical competencies and existing instruments, including those mentioned above, were reviewed by Enders (2011). Regarding the application to medical research learners, these instruments were intended to assess (1) physicians’ ability to read the medical literature and (2) for undergraduate statistics, the alignment with core competencies necessary for the successful use of statistics. Included in this review were widely recognized guidelines for clinical research that are inherently based on statistical concepts; the specific guidelines are the Consolidated Standards of Reporting Trials (CONSORT) document (Moher et al. 2010) which describes fundamental statistical methods for the design, analysis, and reporting of clinical research and lists criteria for writing manuscripts from randomized clinical trials; and the Transparent Reporting of Evaluations with Nonrandomized Designs (TREND) and Strengthening the Reporting of OBservational studies in Epidemiology (STROBE) manuscript guidelines (Des Jarlais et al. 2004; Vandenbroucke et al. 2007; von Elm et al. 2007) which describe criteria similar to those in the CONSORT document for observational studies.

Statistical competencies from the CTS and PH competency documents were combined in order to obtain a full set of competencies for graduate-level biostatistics. The set of statistical competencies was then compared with the set of statistical methods needed to write manuscripts that are listed in the CONSORT, TREND, and STROBE guidelines. The instrument validation included an examination of content validity and criterion validity, which itself included concurrent validity and responsiveness. A comprehensive set of 17 statistical competencies was obtained from the CTS and PH competency documents and the three sets of manuscript guidelines. Eight other statistical assessment instruments, none of which were designed specifically for a population of medical researchers, were then evaluated. The number and percent of items associated with 24 unique statistical topic areas for the various assessment instruments was then determined. Statistical topic areas included in most of these instruments included the following: “selecting assumptions and selecting an appropriate method, unadjusted methods for independent continuous and binary data, significance testing and *p*-values, confidence intervals, and clinical relevance versus statistical significance” (Enders 2011). Finally, a comparison and gap analysis of the 24 statistical topics versus the 17 statistical competency areas was presented. Additional statistical topics that were needed, none of which were included in any of the instruments that were assessed, were also discussed.

The assessment of these competencies and instruments demonstrated that there was a need for a new instrument to assess biostatistical competencies for medical researchers. It was determined that the existing instruments, which included the CTS and PH documents, the CONSORT, TREND, and STROBE guidelines, and eight other statistical assessments, did not sufficiently address the competencies required for medical research using statistics. It was also determined that the set of questions taken across these instruments did not sufficiently address these competencies. In addition, it was determined that a new assessment instrument should be developed and validated by a group of interinstitutional experts in biostatistics as used in clinical research. It was thought that such an instrument could become a definitive list of statistical competencies that are required by medical research learners.

## 3. Development of A New Assessment Tool to Assess Statistical Competencies

The development of the new assessment tool (Oster et al. 2015) began in 2012 through the national CTSA Biostatistics, Epidemiology, and Research Design (BERD) Key Function Committee (KFC). This effort, which continued through 2014, took place via annual Face-to-Face Meetings of the BERD KFC and conference calls. Many of the individuals involved in this effort were members of the BERD KFC Education Working Group and its parent committee, the Online Resources Task Force, although any member of the BERD KFC was welcome to participate.

The new assessment tool was developed around the previously published set of 17 statistical competencies (Enders 2011) described in the previous section. Four new competencies reflecting recent developments in statistical practice were added to this list, bringing the total number of competencies to 21. The goal of this research was to address the following question: “What depth of statistical knowledge do different CTS learners require?”

For three types of CTS learners (principal investigator, coinvestigator, informed reader of the literature), each with different backgrounds in research (no previous research experience, reader of the research literature, previous research experience), 18 experts in biostatistics, epidemiology, and research design proposed levels for the 21 statistical competencies. Each expert was asked for each topic what level of learner competency was needed (none, some, and high) given the type of CTS learner and the research background. Statistical competencies were categorized as fundamental, intermediate, or specialized based on the responses of the experts and according to the previously described criteria for grouping the competencies (Oster et al. 2015).

Statistical competencies that were assessed by the experts appear in Table 1 under “Oster et al. (2015), unless otherwise specified.” Of the 21 statistical competencies that were assessed, nine (43%) were classified as fundamental, six (29%) were classified as intermediate, and six (29%) were classified as specialized. The rank assigned to each of these competencies is based on a subsequent evaluation of these and additional statistical competencies (Enders et al. 2017); this evaluation is described later in Section 4.

Based on the criteria for grouping the competencies, which were then based on the responses about what level of learner competency was needed, it became clear to us that CTS learners who intend to become independent principal investigators require more specialized training, while those intending to become informed consumers of the medical literature require more fundamental education. In addition, we noticed that for most competencies, less training was proposed for those with a higher-level background in research. This led us to conclude that not all of the statistical competencies are appropriate for the goals and perspectives of all learners. We also noted that rather that providing a one-size-fits-all curriculum for statistical learning, institutions should account for the multifaceted needs of different types of learners. Our advice to readers was that tailoring education to baseline knowledge, learner background, and future goals increases learning potential while minimizing classroom time.

We also proposed further changes to our list of statistical competencies. After we had already obtained our results, we noted that some of the fundamental and intermediate competencies had verbs associated with higher cognitive function. After a detailed examination of these competencies, we concluded that some of them would be better phrased using a different verb, which would imply that the goal of the competency would then be different, i.e., to reduce the required competency level for the specific type of learner or to increase the required competency level for a specific type of learner. We then described our specific proposed changes to the wording of several of the competencies (Oster et al. 2015).

## 4. Refinement of the Tool to Assess Statistical Competencies

After a presentation on the previously described statistical competencies to members of the Association for Clinical and Translational Statisticians (ACTStat) and a follow-up discussion with several members of this organization, it became clear that the terminology used to describe the statistical competencies was the basis of the consideration of ACTStat discussants as fundamental to the needs of a medical research learner. These discussants suggested that the competencies should be phrased in a manner that would reflect the fundamental needs of medical research learners. They also suggested that some specific competency areas may be required for some learners. Accounting for this and for the changes that we already proposed to our list of statistical competencies (Oster et al. 2015), a working group was formed in order to revise the competencies so that they would reflect the role of the medical research learner within the research team.

We then evaluated the refined set of statistical competencies for medical research learners (Enders et al. 2017). This revised set now included a total of 24 statistical competencies. The goal of the study was to determine the degree to which each statistical competency is fundamental, i.e., necessary for all learners, or specialized, i.e., necessary for only some learners. Respondents were all doctorally trained biostatisticians and epidemiologists who had taught statistics to medical research learners during the previous 5 years (from 2010 to 2015). These respondents rated the 24 statistical competencies on a semi-quantitative five-point Likert scale anchored by “fundamental” and “specialized.” There were 112 responses.

Statistical competencies that were evaluated appear in Table 1 under “Enders et al. (2017).” Of the 24 statistical competencies that were assessed, 19 (79%) were classified as fundamental and five (21%) were classified as not fundamental. The rank assigned to each of these competencies is based on the percentage of respondents who stated that the competency is fundamental, from highest percentage to lowest percentage. The wording that was modified from the previous assessment (Oster et al. 2015) to this evaluation (Enders et al. 2017) is displayed in bold text.

This research identified a set of 19 statistical competencies that are needed by all medical researchers and five statistical competencies that are needed by only some medical researchers. The different groups of educators from many outstanding academic institutions that were included in the survey all offered a similar perspective, indicating that these results may be viewed as comprehensive for medical research learners. Medical research learners who master the set of fundamental competencies and who engage in team science should have the appropriate statistical skills to be successful in collaborative research. We suggest that these competencies be considered when designing statistical curricula for medical research learners. They should also be used by statistical educators when deciding on which topics will be taught in graduate programs and medical courses where learners need to read and understand the medical research literature.

## 5. The Origin and the Inclusion of Statistical Competency Items

**Figure 1.**
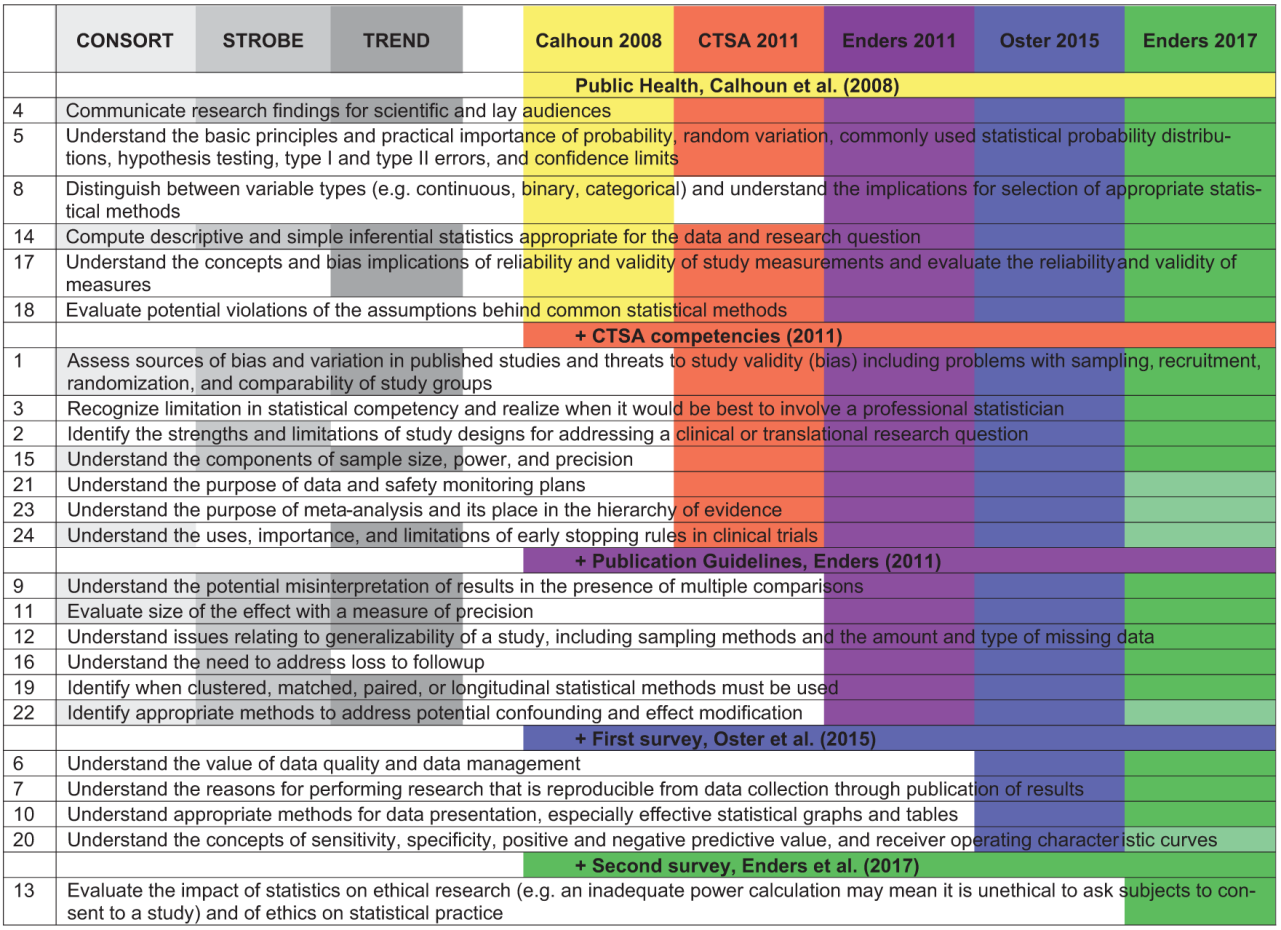
Origin and inclusion of statistical competency items. In gray are competencies identified in Enders (2011) from the publication guidelines (CONSORT, TREND, and STROBE). In color are competencies included for each subsequent publication. For Enders et al. (2017), competencies in light green are those identified as Not Fundamental.

The origin of the statistical competency items and the inclusion of these items into the publications that are cited appear in Figure 1. The competencies identified in Enders (2011) from the CONSORT, STROBE, and TREND guidelines appear in gray. Regarding the other competencies, those included in Calhoun et al. (2008) appear in yellow; those included in the CTSA Education Core Competency Workgroup report (2011) appear in red; those included in the publication guidelines (Enders 2011) appear in purple; those included in the first survey (Oster et al. 2015) appear in blue; and those included in the second survey (Enders et al. 2017) appear in green. The rank of the competency is as described in the previous section.

In Enders et al. (2017), the 19 statistical competencies that were identified as fundamental, i.e., those that are needed by all medical research learners, appear in dark green, and the five statistical competencies that were identified as not fundamental, i.e., those that are needed by only some medical research learners, appear in light green. This is also the only publication that includes an assessment of all of the 24 statistical competencies.

## 6. Potential Uses and Applications of the Statistical Competencies Within Statistics for the Health Sciences

Once we have an understanding of both which statistical competencies are taught to medical research learners and which are not well addressed, there may be opportunities to develop educational modules to fill the gaps. Strong content is resource-intensive to develop and implement; in the era of online learning, such resources need not be spent at each institution. In order to facilitate this, content would need to be modularized and available on an on-demand basis in order to fit in with scheduling at each site. Content would ideally also be available on a tiered basis to provide different levels of intensity to different learners.

Our work has shown that learners who will be principal investigators require a higher level of understanding than those who will be coinvestigators. Institutions should consider assessing learning options to ensure that content is available at different levels, rather than providing a one size fits all approach. In order to do this without expanding the number of courses, learners could access additional course content within existing courses. Such an approach could tie course content to the intended level of each learner determined during the program application phase.

Another way to utilize our work would be to develop assessment tools to assess the statistical competency of learners at matriculation and prior to program completion. The statistical competencies are specific and can be used to develop such assessments that would then be validated prior to use. In practice, some learners have knowledge at the start of their training program and might use such assessments to test out of courses or portions of content, which would constitute a valuable asset in programs in which the majority of learners are practicing clinicians. Assessments could also be used to ensure learner-appropriate competency prior to program completion.

## 7. Implications for Undergraduate Statistics Education

Undergraduate statistics students who are in courses designed for nonmajors may become medical researchers or other health scientists. For this group, the competency work above is begun during their undergraduate training. The revised Guidelines for Assessment and Instruction in Statistics Education College Report (GAISE) (GAISE College Report ASA Revision Committee 2016) is well aligned with the competency work above and now presents an even greater emphasis on providing students with experience with collecting real data. This is well aligned with one of our most fundamental competencies, the need to “assess sources of bias and variation.” While we believe that variation is already well addressed with the GAISE, we believe bias may be underaddressed. Within both flavors of undergraduate courses (statistical literacy and statistical methods), instructors could build upon the GAISE recommendations by introducing the concept of bias especially when students are working with real data. Within the revised college-level GAISE report, bias is included within Recommendation 2 (Focus on conceptual understanding); however, this concept is lacking in Recommendation 3 (Integrate real data with a context and a purpose). We hope that undergraduate instructors will include the concept of bias whenever possible because it is so integral to future understanding of statistical concepts.

## 8. Conclusion

Our work on this topic has progressed from identifying a set of statistical competencies for learners in academic medicine to developing and validating the competencies themselves. Our plan is now to put the competencies to use by assessing the degree to which each is taught in required statistical course-work for learners of different types. We believe this work will help strengthen statistical training to medical research learners and can help enhance undergraduate statistics education.

## Figures and Tables

**Table 1.** Original and revised wording for statistical competencies with results of surveys from Oster et al. (2015) and Enders et al. (2017). Modified wording is shown in bold text.

Rank	Oster et al. (2015), unless otherwise specified		Enders et al. (2017)	
1	Assess sources of bias and variation in published studies and threats to study validity (bias) including problems with sampling, recruitment, randomization, and comparability of study groups	Fundamental	Assess sources of bias and variation in published studies and threats to study validity (bias) including problems with sampling, recruitment, randomization, and comparability of study groups [no change]	Fundamental
2	**Assess** study designs for addressing a clinical or translational research question	Fundamental	**Identify the strengths and limitations** of study designs for addressing a clinical or translational research question	Fundamental
3	**Collaborate with biostatisticians in the design, conduct, and analyses of clinical and translational research**	[Not Assessed]	**Recognize limitation in statistical competency and realize when it would be best to involve a professional statistician**	Fundamental
4	Communicate research findings for scientific and lay audiences	Fundamental	Communicate research findings for scientific and lay audiences [no change]	Fundamental
5	**Assess** the basic principles and practical importance of probability, random variation, systematic error, sampling error, measurement error, commonly used statistical probability distributions, hypothesis testing, type I and type II errors, and confidence limits	Fundamental	**Understand** the basic principles and practical importance of probability, random variation, commonly used statistical probability distributions, hypothesis testing, type I and type II errors, and confidence limits [shortened]	Fundamental
6	**Understand appropriate** data quality and data management **procedures**	Specialized	**Understand the value of** data quality and data management	Fundamental
7	Understand the reasons for performing research that is reproducible from data collection through publication of results	Fundamental	Understand the reasons for performing research that is reproducible from data collection through publication of results [no change]	Fundamental
8	Assess the different measurement scales and the implications for selection of statistical methods to be used on the basis of these measurement scales	Intermediate	**Distinguish between variable types (e.g., continuous, binary, categorical) and understand the implications for selection of appropriate statistical methods**	Fundamental
9	Assess results in light of multiple comparisons	Intermediate	**Understand the potential misinterpretation of results in the presence of multiple comparisons**	Fundamental
10	Understand appropriate methods for data presentation, especially effective statistical graphs and tables	Fundamental	Understand appropriate methods for data presentation, especially effective statistical graphs and tables [no change]	Fundamental
11	**Assess** size of the effect with a measure of precision	Fundamental	**Evaluate** size of the effect with a measure of precision	Fundamental
12	Assess the study sample, including sampling methods, the amount and type of missing data, and the implications for generalizability	Fundamental	**Understand issues relating to generalizability of a study, including sampling methods and the amount and type of missing data**	Fundamental
13	[Developed for this survey]		Evaluate the impact of statistics on ethical research (e.g., an inadequate power calculation may mean it is unethical to ask subjects to consent to a study) and of ethics on statistical practice (e.g., collecting valid data from consenting subjects while maintaining privacy)	Fundamental
14	Assess simple descriptive and inferential statistics that fit the study design chosen and answer research question	Intermediate	**Compute descriptive and simple inferential statistics appropriate for the data and research question**	Fundamental
15	Understand how to determine sample size, power, and precision for comparisons of two independent samples with respect to continuous and binary outcomes	Specialized	**Understand the components of sample size, power, and precision**	Fundamental
16	**Understand statistical methods appropriate** to address loss to followup	Specialized	**Understand the need** to address loss to followup	Fundamental
17	**Assess** the concepts and implications of reliability and validity of study measurements and evaluate the reliability and validity of measures	Fundamental	**Understand** the concepts and bias implications of reliability and validity of study measurements and evaluate the reliability and validity of measures	Fundamental
18	Assess the assumptions behind different statistical methods and their corresponding limitations and describe preferred methodologic alternatives to commonly used statistical methods when assumptions are not met	Intermediate	**Evaluate potential violations of the assumptions behind common statistical methods**	Fundamental
19	Identify inferential methods appropriate for clustered, matched, paired, or longitudinal studies	Intermediate	**Identify when clustered, matched, paired, or longitudinal statistical methods must be used**	Fundamental
20	Characterization of diagnostic testing, including sensitivity, specificity, and ROC curves	Specialized	**Understand the concepts of sensitivity, specificity, positive and negative predictive value, and receiver operating characteristic curves**	Not Fundamental
21	**Defend the significance** of data and safety monitoring plans. [Wording from CTSA, 2011]	[Not Assessed]	**Understand the purpose** of data and safety monitoring plans	Not Fundamental
22	Identify adjusted inferential methods appropriate for the study design, including examination of interaction	Intermediate	**Identify appropriate methods to address potential confounding and effect modification**	Not Fundamental
23	Understand the uses of meta-analytic methods	Specialized	**Understand the purpose of meta-analysis and its place in the hierarchy of evidence**	Not Fundamental
24	**Explain** the uses, importance, and limitations of early stopping rules in clinical trials	Specialized	**Understand** the uses, importance, and limitations of early stopping rules in clinical trials	Not Fundamental
